# Characterisation of complementary feeding practice and locally available climate-resilient crops for complementary food among agro-pastoralists of Ethiopia: a qualitative study

**DOI:** 10.1017/jns.2024.53

**Published:** 2024-09-19

**Authors:** Derese Tamiru Desta, Tadesse Fikre Teferra, Samson Gebremedhin

**Affiliations:** 1 School of Nutrition, Food Science and Technology, Hawassa University, Hawassa, Ethiopia; 2 Institute for Enhancing Health through Agriculture, IHA, Texas A&M University, College Station, TX 77843, USA; 3 School of Public Health, Addis Ababa University, Addis Ababa, Ethiopia

**Keywords:** Agro-pastoralists, Children, Climate-resilient crops, Complementary food, Food group, Moringa, Sorghum

## Abstract

The current study aims to characterise the complementary feeding practice and identify locally available climate-resilient crops that can be used for complementary feeding among agro-pastoralists in Ethiopia. A phenomenological study in Benna-Tsemay district, comprising focused group discussions, key informant interviews, and household observations, was conducted. A pretested guide was used to capture information regarding types of complementary food, lists of food items, and ingredients included in their complementary formulation. A thematic analysis for emerging points of discussion was carried out. Three major themes, including infant and young child feeding practices, food items included in complementary food, and their consumption frequencies, as well as the incorporation of climate-resilient crops into complementary foods as coping mechanisms, emerged. Breastfeeding was common and regarded as essential. Gruel and porridge from grains, roots, and tubers were regular parts of complementary foods in the study area. Moringa and sorghum were dominantly identified as climate-resilient crops regularly grown and used in complementary foods. Growing these crops was regarded as a coping strategy for drought and seasonal constraints. The district is one of the most drought-prone areas in Ethiopia, compromising the quality of complementary food. Unlike the World Health Organization recommendation, the grains, roots, and tubers-based diet formed the basis of complementary food lacking flesh foods, eggs, pulses, and other fruits and vegetables. Thus, it is recommended to improve complementary food quality through value-addition using locally accessible crops.

## Graphical abstract

**Figure:**
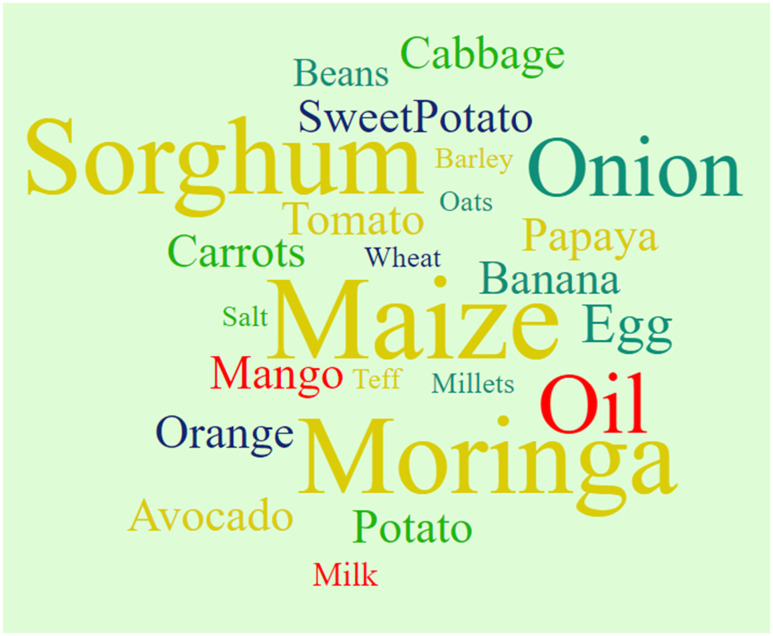

## Background

The World Health Organization (WHO) defines complementary feeding as the introduction of foods and liquids alongside breast milk to meet the evolving nutritional needs of infants exceeding those solely provided by breast milk.^(1)^ The age between 6 and 23 months of infants and young children is a time when they reach a general and neurological stage of development that enables them to be fed on other foods in addition to breast milk.^(1,2)^ While complementary feeding is a universal practice, its implementation across societies is demonstrably shaped by a complex interplay of cultural beliefs, individual caregiver characteristics, and socioeconomic factors.^(3)^

On a global scale, a significant disparity exists in access to minimally acceptable complementary feeding practices, with suboptimal practices prevalent even in high-income households. This highlights a critical concern—the low rates of minimum dietary diversity observed across the world. This is further compounded by insufficient consumption of fruits, vegetables, and animal-source foods in children’s diets. Insufficient quantities and inadequate quality of complementary foods, often due to climate-induced factors, coupled with poor feeding practices, lead to adverse health and nutrition outcomes.^(4)^ These factors particularly impact traditional cereal-based complementary foods, which often lack the required nutrients for growth and development in developing countries like Ethiopia.^(4,5)^ Studies have shown a low prevalence of appropriate complementary feeding practices in Ethiopia, with only 10% of children aged 6–23 months receiving recommended foods and just 12% consuming diversified food groups.^(6,7)^ Several factors contribute to suboptimal practices, including climate change, socioeconomic and demographic status, health service utilisation, individual and household food security, and livelihood status (i.e. pastoralism and agro-pastoralism).^(6–8)^ Agro-pastoral areas in Ethiopia, which home for a significant portion of the population, are particularly vulnerable to climate change impacts.^(9)^ Frequent droughts and high food insecurity prevalence in these communities pose major challenges to child nutrition.^(10,11)^ As a coping mechanism, communities in southern Ethiopia have developed strategies utilising less preferred yet climate-resilient crops.^(12)^

Research in Benna-Tsemay district suggests that complementary feeding practices are heavily influenced by cultural beliefs, traditional knowledge held by women, and prevailing social norms. A study done by Anteneh (2018) highlights that the introduction of complementary foods is typically delayed until the infant reaches six months of age. This delay is often attributed to the fear of abdominal cramps, locally referred to as ‘Gore’ a term designated to the timeframe for the introduction of complementary food. It represents a culturally-embedded method for assessing an infant’s readiness for complementary feeding. Traditionally, a thin, local stick is used to measure the circumference of the infant’s neck and right arm. This practice serves as a marker for determining the appropriate time to introduce complementary foods.^(13)^ Despite this good practice, cereals dominate homemade complementary foods, with sorghum being a common constituent in various forms like gruel, porridge, fetfet, kitta, and dabo.^(14,15)^ Sorghum and moringa leaves are identified staple crops in Benna-Tsemay.^(13)^

However, a systematic investigation into their utilisation within complementary feeding practices is currently lacking. This study intends to characterise the complementary feeding practices among agro-pastoral communities and identify locally available climate-resilient crops as part of an effort to improve complementary foods based on locally available resources. In this regard, this research will contribute to the body of knowledge on complementary feeding practices and climate-resilient crops. Characterising both is crucial for several reasons. In Ethiopia, particularly the Benna-Tsemay district, exemplifies the importance of this research. Many traditional crops, with the potential to significantly enrich the nutrient profile of complementary foods, have been overlooked in favour of staple crops. Characterisation of these underutilised crops can unlock their hidden potential, promoting their use in complementary feeding interventions. Additionally, with climate change disrupting traditional growing seasons and weather patterns, characterisation becomes a vital tool for identifying crops that can adapt to these changes. This, in turn, can ensure food and nutrition security, thereby reducing the risk of malnutrition among children aged 6–23 months in agro-pastoralist communities.

## Methods

### Study setting

**Fig. 1.**
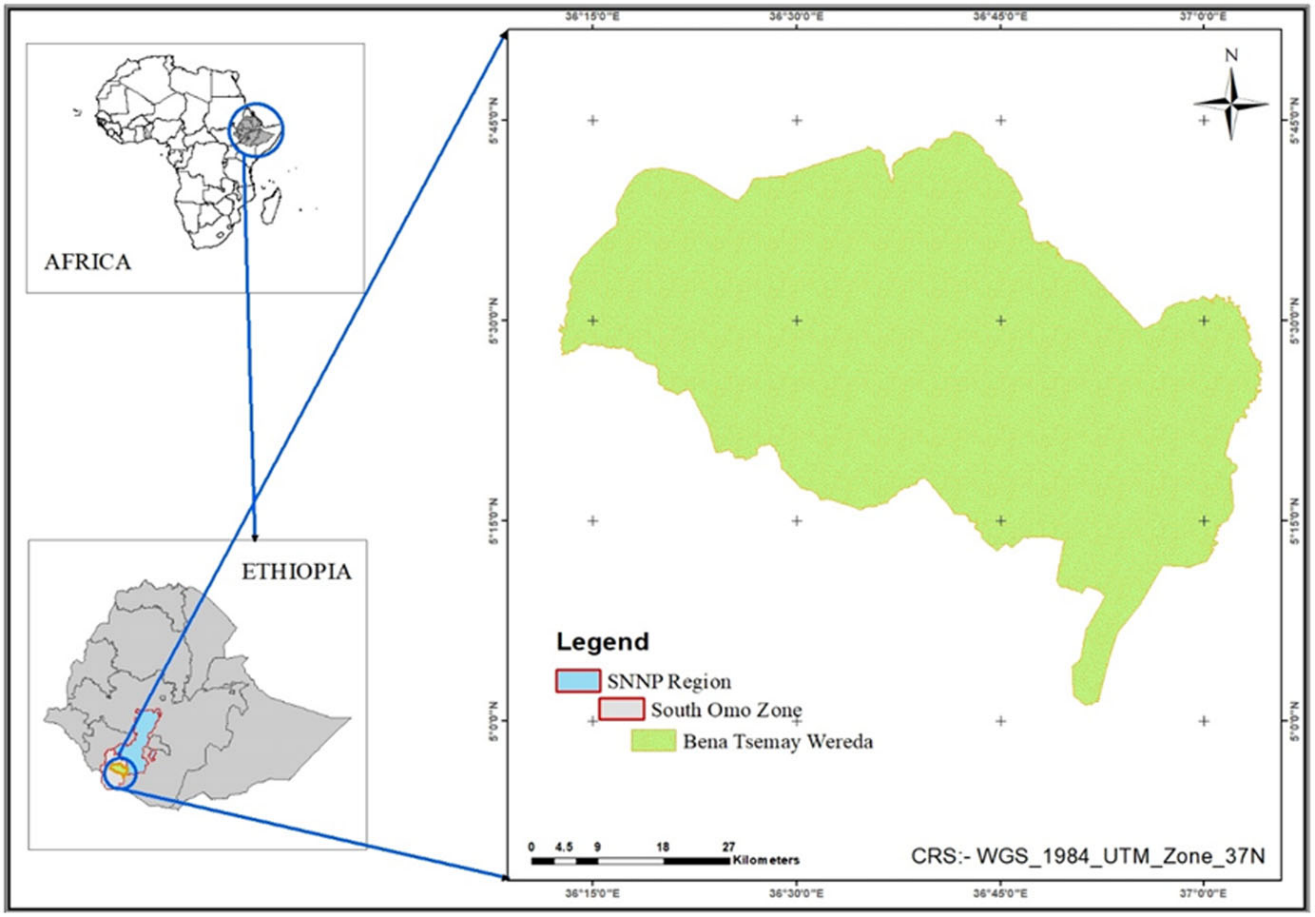
Map of Benna-Tsemay District, South-Omo Zone, Ethiopia (2024).

The study was conducted among agro-pastoralist communities of Goldia and Buneker sub-districts in the Benna-Tsemay district of South-Omo zone. The zone is one of the administrative units of the former Southern Nations, Nationalities, and Peoples’ Region of Ethiopia and is currently structured under the South Ethiopia Regional State (Fig. 1). It is situated in the southwestern part of Ethiopia, bordering the Omo River to the east Bench Maji to the south and west, respectively. Benna-Tsemay is one of the five agro-pastoral districts in the South-Omo zone. It is located 739 kilometres in the south of Addis Ababa, the capital city of Ethiopia. It is composed of 35 sub-districts under six clusters, and its total population was 76,647.^(16)^ The district is 1500 metres above sea level, and its average yearly temperature ranges from 26 °C in the winter to 40 °C in the summer. With an average annual rainfall of 800 mm, the distribution of rainfall is bimodal. The district has a lengthy dry season from December to the start of March, followed by a short dry season in June and July.^(17)^ In this district, mixed farming is the main livelihood of the population, where agrarians and agro-pastoralists reside evenly across the clusters.^(18)^ The major agricultural products include moringa tree (*Moringa stenopetala and Moringa oleifera*), sorghum, maize, pearl millet, teff, common bean, sesame, cowpea, sunflower, sweet potato, mango, papaya, avocado, banana, cabbages, tomato, pumpkin, and others.^(19)^

### Study design and participants

Two agro-pastoral sub-districts were selected by stratified purposive sampling due to their larger population size and administrative clusters in the district and were included in an in-depth descriptive phenomenological study. Ten key informant interviews, nine overt types of direct household observations, and two focused group discussions were conducted from December 11 to 27, 2022. The participants for the focused group discussion and direct household observations comprised women with young children aged between six and twenty-three months. The district coordinator of the health office, public health expert, nutrition focal person, agriculture experts, agricultural researchers, health extension workers, development agents, and social and behaviour change communication (SBCC) experts in the district were among the key informants who were interviewed. The key informant profile is summarised and presented in the supplementary material.

### Data collection procedures and techniques

A pretested guide was used for both focused group discussions and key informant interviews. The guides are included in the Appendix 2 of Supplementary Material. The guides focus on feeding practices, including breastfeeding and the introduction of complementary foods, types of foods available as complementary foods, types of locally available and climate-resistant seasonal crops, frequency of food items consumed by the children, and challenges associated with complementary feeding. Climate-resilient crops were defined as any crops mentioned by the study participants. Of the lists, crops were termed climate-resilient crops, which have enhanced tolerance to biotic and abiotic stresses. Specifically, crops that adapt to diminishing crop yields in the face of droughts, higher average temperatures, and other climatic conditions were termed climate-resilient crops.^(20)^ The food groups considered in this study were the seven initially recommended by the WHO and the United Nations Children Fund (UNICEF). The food groups were grains, roots, and tubers (1); pulses (2); dairy (3); flesh (4); eggs (5); vitamin A-rich fruits and vegetables (6); and other fruit and vegetables (7).^(1)^

Furthermore, an observation checklist was developed to acquire information about complementary food preparation and feeding to triangulate the focused group discussion and key informant interviews. The direct observations were aimed at achieving triangulation of discussion points raised during the focused group discussions, and identification of commonly included food items in the complementary foods. The observation checklist includes crucial questions about complimentary food types, lists of food items, and ingredients.

The key informant interviews were done in a separate district health and agricultural office rooms to ensure the interviewee’s privacy. The individual interviews with key informants lasted approximately forty minutes each. The two focused group discussions were held at the sub-district health posts. The first and second focused group discussions lasted for two and a half hours and two hours, respectively. Whereas, each direct observation session lasted for one and a half hours. The principal investigator led all data collection activities, including key informant interviews, household observations, and focused group discussions. To ensure effective communication with participants, the principal investigator was accompanied by an experienced nutritionist and public health expert fluent in Benna and Tsamai, the two local languages. Data saturation for each data collection method (focused group discussions, direct household observations, and key informant interviews) was achieved when point of discussion redundancy in the collected data. The redundancy was characterised by discussions reaching a point where new insights became scarce, and recurring discussion points dominated.

### Data processing and analysis

Thematic analysis was performed in six steps.^(21)^ During the initial phase, all interviews were audio- and video recorded. The second phase involved preparing and organising data for analysis using verbatim transcripts. Verbatim transcription and translation were done by a public health expert who speaks and listens in the local language. In the third phase, the transcriptions were reviewed for gaps and limits. In the fourth step, evolving data and potential biases were evaluated. In the fifth step, all evolving data was coded and categories were created. Manual coding was employed to analyse the qualitative data collected through the three methods: focus group discussions, key informant interviews, and direct household observations. To ensure reliability and consistency throughout the coding process, several strategies were implemented. Firstly, the coding outline was developed, aligning with the research question that centred on complementary feeding practices and the inclusion of climate-resilient crops into complementary foods. It encompassed specific codes such as breastfeeding practices, timing of complementary food introduction, types of food and ingredients used, seasonal availability of crops, and challenges encountered during complementary feeding (Table 1). Secondly, memoing served as a valuable tool. The documented detailed written notes during the focused group discussions, key informant interviews and direct household observation alongside assigned codes. These memos captured their reflections, interpretations, and emerging connections between various data segments. This practice enriched and deepen understanding of the emerged subthemes from the three of the qualitative data collection methods. Finally, any discrepancies or disagreements in coding decisions were addressed through ongoing refinement of the coding scheme. This process ensured consistency and reliability in the analysis.

**Table 1.** Examples of themes and subthemes emerged with their respective codes and meanings

Themes	Subthemes	Codes	Meaning or definitions
Infant and young child feeding practices	Benefits of breastfeeding	Health and Immunity	Breastfeeding helps protect babies from illnesses and infections. *….breastfeeding is good for my child’s health and protects from repeated diseases.*
		Bonding and Development	Breastfeeding fosters a close emotional connection between mother and child. *I feel great happiness when I breastfeed and it is good for my child’s physical strength.*
	Complementary foods introduction	Current Practices and perceived health benefit	Untimely introduction of complementary food. *Children who begin before or lately six months are frequently sick. They commonly have diarrhoea, vomiting, weight loss, and gastrointestinal distress, while children who start at six months are healthier and grow faster*
	Foods group	Food ingredients	Food used to prepare gruels, porridges, and bread for children. *I add a variety of foods to my child’s diet, including grains like sorghum, corn, millet, wheat, oats, teff, and barley, as well as legumes such as Yergib ater pigeon pea, peas, lentils, and kidney beans.*
Climate-resilient crops in complementary foods	Climate-resilient crops as coping strategies	Seasonal Availability	Fluctuations in the availability of certain food items throughout the year. *Sorghum (locally called alaffa), millet, cassava, and sweet potatoes are accessible year-round. If it is not available, we can purchase it on the market or borrow it from someone.*
Existing challenges	Challenges associated with complementary feeding	Farming Activities	Tasks related to agricultural activities. *…women have to engage in agricultural activities, including clearing weeds, harvesting crops, cultivating vegetables, and selling cash crops.*
		Social Obligations	Participation in community events. *…even if women breastfeed, they are responsible for social events, such as burial rituals and wedding.*
		Food Availability	Fluctuations in food availability due to seasons. *The community is almost aid dependent for food shortages caused by drought and less agricultural productivity.*

Finally, during the six phases, comparable subthemes were organised into distinct themes. All related subthemes were categorised into three themes: infant and young child feeding practices, food items included in complementary food and their consumption frequencies, as well as the incorporation of climate-resilient crops into complementary foods as coping mechanisms.

### Rigor and trustworthiness of the study

In order to establish the credibility and trustworthiness of the research, three qualitative methods, including focused group discussions, direct household observations, and key informant interviews, were utilised to gather data. Finally, the data collected from these diverse sources was then triangulated to ensure accuracy and validity. In addition, to strengthen the credibility of the study, the researcher employed a saturation sampling strategy. This involved prolonged engagement with participants across the various qualitative data collection methods. In that case, the data collection continued until data saturation level was achieved. To ensure the transferability of the study findings, the researcher conducted a comprehensive literature review of existing research on complementary foods in Ethiopia. This informed the development of all data collection tools, grounding them in established knowledge. The study site was purposefully chosen based on livelihood characteristics, and participants were selected to ensure they were representative of the district population. To strengthen the dependability of the research findings, a process of triangulation was employed. This involved comparing the results obtained through all the qualitative data collection methods with findings from previously published research on complementary foods in Benna-Tsemay district, and other agro-pastoralist communities in Ethiopia. This cross-checking aimed to identify agreement and disagreement between the current study and existing literature, enhancing the consistency and trustworthiness of the research.

The study also employed a triangulation approach, integrating data from focus group discussions (FGDs), key informant interviews (KIIs), and direct household observations to gain a comprehensive understanding of the complementary feeding practices and inclusion of climate-resilient crops. A core finding emerged through convergence across all three data sources. For instance, all participants from the Focused Group Discussions (FGDs), key informants (KIIs), and household observations confirmed the use of cereals (maize, sorghum, wheat, millet, teff) and moringa leaf as primary components of complementary foods. This convergence strengthens the conclusion that cereals form the major focus of complementary feeding practices within the study area. On the other hand, the divergences between data sources provided further insights. For instance, compared to the KIIs’ emphasis on key ingredients in porridge, the FGDs revealed a wider variety of foods used in complementary feeding. This suggests that while cereals dominate, some households might incorporate additional ingredients based on individual practices and preferences. In this regard, the additional ingredients were identified from the direct household observations. Based on convergence and divergence, sorghum, maize, and wheat can be identified as the staple grains used in most complementary foods, supported by all data sources. Household observations further revealed the incorporation of additional ingredients like teff, pigeon peas, eggs, vegetables, milk, oil, and salt.

While the first author conducted the majority of the data collection, he was assisted by a qualified nutritionist and public health expert. Both experts have more than ten years of experience in community outreach programmes. A half-day training on the overall objective and research questions of the qualitative data collection tools was given to the public health expert and nutritionist. In this regard, the first author as a researcher, mainly interested in infant and young child feeding, previous experience of reviewing research papers may have influenced the expectations of the interviews and discussions. Consequently, the first author expected that the infant and young child feeding practices in the study area would be similar to those in other Ethiopian districts. In the first focused group discussion, the researchers learned that moringa leaf was a part of complementary food, which was not well documented to their knowledge. The crop is well known for its health preferences, specifically for non-communicable diseases among adults. Thus, the researchers had to include probing questions about moringa and other underutilised crops. Then, more probing questions were added to the guides and other qualitative data collection approaches (i.e. household observation). Initially, the key informant interviews using the guide were prepared in English and translated into Amharic (the Ethiopian national language), making it easy to interact with the interviewees from the district health office. However, it was difficult to do the same with the other informants at the sub-district level (locally called kebele). Then the researchers were able to recruit a translator in addition to others to accompany and help translate all the questions and responses into the local languages. This helped to probe the questions and detail the responses. The focused group discussions were held in the local language. All the observations were also done with the help of translators. The role of the first author at the time of the discussions was to take a summary note at the end of the discussion and later triangulate during the narration of the transcribed data. Any thoughts that arose were noted down. Prior to the focused group discussions, the translator and the first author had a comprehensive conversation about what to do. All reported responses after the transcription were provided to the transcriber for any biases and crosschecked for additional thoughts. Several statements from the key informant interviews were checked by asking the interviewee on the phone. All other researchers took part in designing the guides and oversaw the whole data collection, analysis, and write-up.

### Ethical clearance

This study was conducted according to the guidelines laid down in the Declaration of Helsinki and all procedures involving human subjects/patients were approved by the Hawassa University’s Institutional Review Board (IRB/394/15). The study followed the essential requirements for working with human subjects. Prior to data collection, district administrators were approached with an explanation of the study and its objectives, and their agreement was obtained. The nature of the study was thoroughly described to the respondents. Participants provided written informed consent to willingly participate in the study, and they were assured that they may opt-out or quit at any moment. Furthermore, confidentiality of participant’s information was assured and information was recorded and published anonymously. Those who are practicing inappropriate complementary feeding were advised to improve the complementary feeding practice.

## Result

### Socio-demographic characteristics of the study participants

In the current study, twenty mothers participated in the focused group discussion. The participants were all married, and half of them (50%) had never attended any kind of school. A total of ten key informants participated. These informants represented various governmental and non-governmental organisations (NGOs) engaged in diverse nutrition-related activities. The characteristics of the focus group discussion participants and key informants can be found in Tables 1 and 2 of the supplementary material.

Employing thematic analysis from the three qualitative data collection methods, the current study identified three core themes that captured the range of participant experiences: infant and young child feeding practices, the diversity of ingredients used in complementary foods and their feeding frequency, and lastly, the role of climate-resilient crops in complementary foods as coping strategies for addressing food security challenges.

### Infant and young child feeding practices

Participants in the focused group discussion were asked about the key infant and young child feeding practices, such as breastfeeding experience and benefits, exclusive breastfeeding, and the introduction of complementary foods to their infant and young child, and emphasised the satisfaction it provided for them and their child, as well as the numerous health benefits of breastfeeding. In this sense, all of the participants stated that they had experienced breastfeeding. All participants viewed breastfeeding as beneficial to children because it avoids illness and brings pleasure to both infants and breastfeeding women.

*‘I feel great happiness when I breastfeed my child because it is good for my child’s health and physical strength and protects him from repeated diseases’,* says a 32-year-old mother from Buneker sub-district.

Additionally, breast milk was thought to be more contaminant-free than water, making it simple to feed and enjoy breast milk alone until the child reached six months.

*‘We are always advised to exclusively breastfeed for up to six months because breast milk is cleaner than water, easy to feed children, and does not require any additional knowledge to prepare’,* said 28-year-old mothers from the Goldia sub-district.

Furthermore, in the focused group discussion, participants emphasised that exclusive breastfeeding for the first 6 months provides essential nutrients and antibodies that help strengthen the child’s immune system.

A 25-year-old mother from the Buneker sub-district stated, while comparing the early and late introduction of complementary food, that *‘children who begin before or lately six months are frequently sick. They commonly have diarrhoea, vomiting, weight loss, and gastrointestinal distress, while children who start at six months are healthier and grow faster’*

The participants reflected on their experience with the introduction of complementary. They believed complementary food should be introduced at six months of age due to several reasons. They noted that giving complementary food too soon or late might raise the risk of infection in infants and young children.

*‘Now, we start complementary feeding at six months because we understand the benefits of breast milk’,* said a 31-year-old mother from Buneker sub-district.

### Diversity of ingredients in the complementary foods and feeding frequency

During the focused group discussion, participants conveyed that gruel and porridge are commonly regarded as complementary foods tailored for the nutritional needs of infants and young children. The primary components typically incorporated into these complementary foods encompass maize, sorghum, wheat, millet, moringa leaf (referred locally as Aleko), teff, pigeon pea (referred to locally as ‘yergib ater’), eggs, cabbage, cow’s milk, onions, oil, and table salt.

A participant, a 33-year-old mother residing in the Buneker sub-district, listed a variety of commonly available food items incorporated into her child’s diet. *‘I add a variety of foods to my child’s diet, including grains like sorghum, corn, millet, wheat, oats, teff, and barley, as well as legumes such as Yergib ater (pigeon pea), lentil, and kidney beans’*

**Fig. 2.**
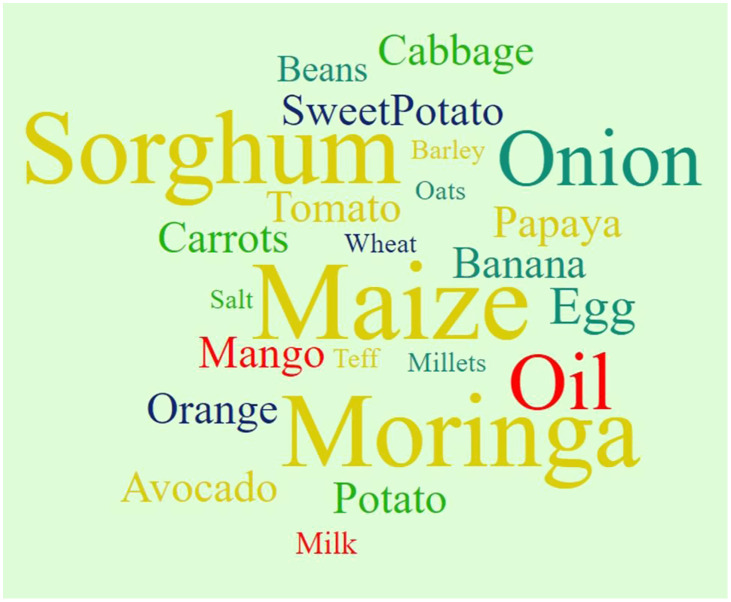
Ingredients in the complementary foods. The larger size of the words indicates the higher frequency mentions during the focused group discussions and observed during direct household observations (December 2022).

During household observations, these foods were also used in complementary food. Lists of food items and ingredients observed are described in Fig. 2 and Table 3 of the supplementary material.

Maize, sorghum, teff, millet, and wheat were popular food items from the grains, roots, tubers, and plantains food group. Kidney beans, lentils, peas, and pigeon peas were from the pulse, nut, and seed food groups. Both of these food groups were the most often provided, as indicated by the participant. Only moringa leaf, carrot, tomato, papaya, and mango were identified as vitamin A-rich fruits and vegetables. According to the participants, the intake of this food group is determined by its availability in homes as well as the season, but moringa leaf, a vitamin A-rich fruit and vegetable, was readily available and incorporated into the complementary food.

A 25-year-old mother from Buneker further elaborated on the food items that she incorporates into her child’s diet. *‘I also include vegetables in my child’s diet, such as cabbage, onion, tomatoes, aleko (moringa leaf), and carrot’.*

Cow and goat milk were the dairy food categories accessible and given to infants and young children. According to the participants, these food products remain dependent on household availability. Yet again, the egg was available once a week, based on the family’s capacity to purchase it. The flesh food group was the least given, with only once a month provided to those who could afford it. In addition to the aforementioned food groups, oil and table salt were frequently included in complementary foods.

A 30-year-old mother in Goldia added animal-source foods, supplementing the previously mentioned items. However, she noted that consumption patterns were heavily influenced by availability. *‘We do have access to some animal products like milk, egg, and oil. Between meat and butter, it depends. If we have cows and chickens available, we might get to have meat or eggs once a week, but that’s only affordable for some families. As for meat and butter in general, those might only be consumed once a month, or even just once a year’*

### Climate-resilient crops in complementary foods, coping strategies, and challenges

Participants reported that they are using climate-resilient crops as coping mechanisms for food insecurity caused by seasonal variation. According to the participants in the focused group discussion, the most climate-resilient crops were sorghum, millet, and moringa leaf, all of which are common constituents of complementary foods. All focused group discussion participants and key informants stated that, compared with others, these crops are accessible throughout the year. During the household observations, the majority of women included boiled moringa leaf and sorghum flour in the complementary food. An example of blended maize, sorghum and moringa leaf as a complementary food for an eleven-month-old child is depicted in Fig. 1 of supplementary material.

In addition, during the focused group discussion, participants addressed a variety of problems linked to complementary food. The primary challenges are recurrent droughts and the seasonality of products used in complementary food. Workloads connected to home duties, social duties, and tough agricultural operations create time limits for preparing and serving complementary food for their respective infant and young children.

*‘In our community, women have to engage in agricultural activities, including clearing weeds, harvesting crops, cultivating vegetables, and selling cash crops. These activities are done outside the home, which may hinder us from timely preparation of complementary food and feeding our children’.* A 28-year-old woman from Buneker sub-district mentioned, and all other focus group participants agreed.

A 30-year-old mother from Goldia sub-district added *“…even if women breastfeed, they are responsible for social events, such as burial rituals, which may occur outside of the house for an extended period of time and pose a difficulty’’*

Furthermore, the key informants underscored many issues that the study district is experiencing. Low food production caused by drought and seasonal variation is posing challenges for the community. A monotonous diet consisting primarily of cereal, along with water shortages, is common.

*“The community is almost aid dependent for food shortages caused by drought and less agricultural productivity. Mainly porridge from sorghum, maize, and wheat is the usual complementary food, which made the complementary feeding practice suboptimal in the study area’’* said the nutrition focal person from Health Office of Benna-Tsemay District.

Furthermore, both focused group discussion participants and key informants reported cultivating drought-resistant crops such as sorghum (locally known as Alaffa), cassava, and moringa trees (locally known as Aleko), with millet as the primary coping strategy for drought and seasonal food scarcity. Other coping mechanisms highlighted throughout the discussions included borrowing from friends and relatives and purchasing low-cost alternative food items from marketplaces.

*“Sorghum (locally called alaffa), millet, cassava, and sweet potatoes are available year-round. If one of these becomes unavailable, we can buy it at the market or borrow it from someone”* A 28-year-old mother from the Buneker sub-district.

## Discussion

This research characterises the diversity of food groups in complementary foods for children aged 6–23 months and explores the use of climate-resilient crops. The thematic analysis revealed three core themes: infant and young child feeding practices, food items and their consumption frequencies of complementary foods, and the strategic use of climate-resilient crops in complementary foods as adaptation mechanisms. These themes underscore the need for quality improvement in moringa and sorghum-based complementary foods. Specifically, the inclusion of other food groups recommended by the WHO—such as flesh foods, eggs, pulses, vitamin A-rich fruits, and vegetables—is critical to address potential dietary inadequacies. Furthermore, the study highlights the ongoing challenges faced by the community, including frequent droughts, seasonal food insecurity, and the significant workload shouldered by women in both household and agricultural tasks.

The WHO recommends a variety of nutrient-rich, safe, and suitable complementary foods for young children, including five out of eight food groups along with breast milk, to ensure adequate macro- and micronutrient intake for healthy growth. The recommended food groups for complementary food include (i) grains, roots, and tubers; (ii) pulses; (iii) dairy products; (iv) meat; (v) eggs; (vi) vitamin A-rich fruits and vegetables; and (vii) other types of fruits and vegetables.^(1)^ Despite the recommendations, the practice of complementary feeding in many developing countries, including Ethiopia, often falls short of these recommendations. For instance, the predominant use of grains, roots, and tubers in complementary foods has been observed in various studies.^(4,15,22–24)^ The current study found that grains, roots, and tubers were the dominant or staple food groups used for complementary foods. This food group offers substantial health benefits since it contains calories, protein, fibre, and minerals.^(1,25)^ This food group is recommended as a part of complementary foods for children aged 6–23 months but other food groups should be complemented. It is also important to choose better high digestibility varieties of cereal grains as the predominant one (sorghum) generally has proteins with poor digestibility and bioavailability, particularly in cooking.^(26)^

Flesh foods and eggs should also be eaten by infants and young children as often as possible. The consumption of these food groups is associated with increased intakes of energy, essential amino acids and fatty acids, vitamin B12, vitamin D, zinc, and other nutrients.^(2,27,28)^ Livelihood contributes to high consumption of Animal Source Foods due to increased access specifically among the pastoralists in Ethiopia,^(29)^ however, the current study confirmed during the direct household observations that the complementary food lacks meat, fish, and eggs, requiring urgent attention.

Food items categorised as legumes and pulses are sources of dietary protein where consumption of animal protein is limited due to various factors. When combined with cereals, they provide well-balanced essential amino acids and are quite important for children.^(30)^ However, none of the women included pulses in the complementary food during the direct household observations in the current study.

The current study further notes a significant gap in the inclusion of vitamin A-rich fruits and vegetables with exception of moringa leaf in complementary foods. The consumption of this food group is vital but is alarmingly low in Ethiopia, as corroborated by several studies.^(6,8,15,23,24,31–35)^ This gap may pose a significant risk of inadequacy of micronutrients, including vitamin A and others. This in turn, potentially, may lead to increased susceptibility to communicable and non-communicable diseases.^(36)^ The long-term impact of low consumption of micronutrient-rich food groups may also contribute to higher mortality risks in adulthood.^(37)^

The current study emphasises the utilisation of climate-resilient crops in complementary foods, a critical consideration in the context of global climate change. To mitigate the effects of climate change and extreme weather events, households in other parts of Ethiopia have adopted coping strategies, notably the consumption of climate-resilient crops.^(25,38)^ The current study confirms utilisation of climate-resilient crops as an approach for complementary foods, particularly moringa and sorghum, which are the main ingredients in complementary food preparations.

Moringa tree is known for its adaptability to extreme weather conditions including high temperatures and rainfall.^(19)^ It is nutritionally rich in vitamins, minerals, and amino acids, making it an accessible food source in developing countries.^(39)^ Despite its benefits, it is sometimes considered a ‘famine food’ due to its year-round availability.^(40)^ The current study also confirmed that moringa leaf is a year-round food source used during droughts and is easily accessible.

While the nutritional benefits of moringa, particularly in reducing micronutrient deficiencies in children, are well documented,^(41–46)^ the methods of cultivation, processing, and storage significantly influence its nutrient content. Boiling was the primary method of processing moringa leaf for complementary food observed during the direct observation, demanding further investigation into its impact on nutrient composition and acceptance by children. Additionally, concerns about the potential reduction in nutrient bioavailability due to anti-nutritional factors present in moringa, especially when processed by boiling, have been raised.^(47)^ On the other hand, moringa leaf-added complementary foods may contain higher levels of some micronutrients, including iron and zinc, which are associated with upper-tolerable intake among under five children,^(42)^ necessitating careful intake monitoring.

Similarly, sorghum is recognised for its resilience to climate variability and water scarcity.^(48)^ It is crucial for food security in arid and semi-arid regions globally^(49)^ and is a staple in Ethiopia, used in various traditional food preparations.^(50,51)^ Although a vital ingredient in traditional grain-based complementary foods for infants and young children,^(14)^ the comprehensive nutritional profile of sorghum is subject to variations due to biotic and abiotic factors.^(51)^ Its low digestibility, particularly in protein content, has led to its perception as a low-value crop for food uses compared to other cereals.^(52)^ The study underscores the need for further research to investigate the impacts of including sorghum in complementary foods.

## Implications of the study

This study highlights several key implications for improving complementary feeding practices within the study area. While the introduction of complementary foods is a positive step, the lack of recommended food groups from the WHO is concerning. These missing groups include flesh foods, eggs, pulses (legumes), vitamin A-rich fruits, and vegetables. This limited diversity suggests a need for interventions that promote the incorporation of these vital food groups into complementary foods. The study identifies sorghum and moringa leaf as core components of complementary foods. However, further research is needed to explore how the inclusion of the missing WHO food groups could improve the overall nutritional value of these moringa and sorghum-based complementary foods. Importantly, the focus on climate-resilient crops presents a valuable strategy for promoting sustainable complementary feeding practices. However, the implications of these crops on the overall nutritional and anti-nutritional content of local complementary foods require further analysis. The study also highlights challenges such as frequent droughts, seasonal food scarcity, and the heavy workload of women in agricultural tasks. These factors contribute to food insecurity and potentially hinder the consistent use of diverse food groups in complementary foods. Therefore, gender-sensitive agricultural interventions are crucial to address these challenges.

This study has a number of limitations and strengths. First, data collection during the non-harvesting season may not fully capture the impact of seasonal variations on food availability in the study area. This potentially limits the generalizability of the findings regarding seasonal food access and its influence on complementary feeding practices. Second, the study’s focus on a single district, despite the region’s diverse ethnic backgrounds, restricts its representativeness of all Ethiopian agro-pastoral communities. Further research across multiple districts with varying ethnic compositions could provide a more comprehensive understanding of complementary feeding practices in Ethiopia. At the same time, the strength of the current study is the inclusion of diverse respondents beyond mothers (key informants from governmental and NGOs) strengthens the study by enriching the data with a broader range of perspectives on complementary feeding practices within the community. This triangulation of viewpoints enhances the comprehensiveness and credibility of the research findings.

### Conclusion

Complementary feeding in the study area primarily relied on gruel and porridge made from grains, roots, and tubers. Notably, moringa and sorghum proved to be the dominant climate-resilient crops cultivated and incorporated into complementary foods. The participants identified the cultivation of these crops as a key coping strategy employed to manage drought and seasonal food limitations. Despite WHO recommendations, complementary food in the study area is still inappropriate in terms of food group composition. Thus current study highlights the need for interventions to promote the inclusion of additional food groups recommended by WHO (flesh foods, eggs, pulses, vitamin A-rich fruits and vegetables) in moringa and sorghum-based complementary foods. This diversification can address potential dietary inadequacies and improve the nutritional quality of complementary feeding practices. Droughts, seasonal food insecurity, and women’s workload, agricultural, and social duties all make it difficult to prepare and feed complementary foods to children on time. This can help to optimise the use of these crops for improved child nutrition outcomes. On the other hand, the study underscores the challenges faced by women, who shoulder a significant workload in both household and agricultural tasks. Gender-sensitive agricultural interventions are crucial to support women and empower them to participate more effectively in food production for complementary food. Finally, the study suggests a need for further research to explore the impact of using climate-resilient crops on the nutritional and anti-nutritional content of complementary foods across multiple districts with varying ethnicities and ecological zones of Ethiopian agro-pastoral communities.

## Supporting information

Desta et al. supplementary material Desta et al. supplementary material

## Abbreviations

**CLIFOOD:** Climate Change Effects on Food Security; **FGD:** Focus Group Discussion; **IRB:** Institutional Review Board; **KII:** Key Informant Interview; **NGO:** Non-Governmental Organization; **SBCC:** Social and Behavior Change Communication; **SDG:** Sustainable Development Goal; **UNICEF:** United Nations Children Fund; **UOHO:** University of Hohenheim; **WHO:** World Health Organization.

## Supplementary material

The supplementary material for this article can be found at https://doi.org/10.1017/jns.2024.53
