# Supplementary material for: Characterisation of complementary feeding practice and locally available climate-resilient crops for complementary food among agro-pastoralists of Ethiopia: a qualitative study
Source: J Nutr Sci. 2024 Sep 19;13:e39. doi: 10.1017/jns.2024.53 (PMC11428050; doi:10.1017/jns.2024.53)
Supplement: Desta et al. supplementary material [file S2048679024000533sup001.docx]

Appendix 1

Table 1: Characteristics of the focused group discussion participants (n=20), Dec. 2022

| Variables | Number | Percentage |
| --- | --- | --- |
| Sub-districts |  |  |
| Bunekar | 12 | 60 |
| Goldia | 8 | 40 |
| Educational status |  |  |
| No formal education | 10 | 50 |
| Primary | 9 | 45 |
| Secondary and above | 1 | 5 |
| Marital status |  |  |
| Single | - | - |
| Married | 20 | 100 |
| Divorced | - | - |
| Widowed | - | - |

Table 2: Summary of key informant information (n=10), Dec, 2022

| S.No | Key Informant Institution | Role | Number interviewed |
| --- | --- | --- | --- |
|  | District Health Office | District Health Office Manager | 1 |
|  | District Health Office | Public Health Expert | 1 |
|  | District Health Office | District Nutrition Focal Person | 1 |
|  | District Health Office | Health Extension Worker | 2 |
|  | District Agriculture Office | District Agriculture Manager | 1 |
|  | Sub-district Agriculture Office | Development Agents | 2 |
|  | Non-Governmental | Nutrition Social and Behavior Change Communication Expert | 1 |
|  | Southern agricultural research institute | Food Science and Nutrition, Associate Researcher | 1 |

Table 3: Summary of observation (n=9)

| Observant number | Sub-districts | Child age(Months) | Type of complementary food | Ingredients included |
| --- | --- | --- | --- | --- |
|  | Goldia | 10 | Gruel: Semisolid, | Moringa leaf, Maize flour, Onion, Oil, and salt |
|  | Goldia | 15 | Gruel: Semisolid, | Moringa leaf, Maize flour, Onion, Oil, and salt |
|  | Goldia | 18 | Gruel: Semisolid, | Moringa leaf, Maize flour, Sorghum flour, Egg Onion, Oil, and salt |
|  | Goldia | 20 | Gruel: Semisolid, | Moringa leaf, Maize flour, Sorghum flour, Egg, Onion, Oil, and salt |
|  | Buneker | 20 | Gruel: Semisolid, | Moringa leaf, Maize flour, Sorghum flour, Onion, Oil, and salt |
|  | Buneker | 13 | Gruel: Semisolid, | Moringa leaf, Maize flour, Sorghum flour, Onion, Oil, and salt |
|  | Buneker | 22 | Gruel: Semisolid | Cow milk, Moringa leaf, Maize flour, Sorghum flour, Onion, Oil, and salt |
|  | Buneker | 19 | Gruel: Semisolid | Sorghum flour, Maize flour, Onion, Oil, and salt |
|  | Buneker | 20 | Porridge | Sorghum flour, Maize flour, Onion, Oil, and salt |

Appendix 2

Focus Group Discussion (FGD) Facilitator Guide, Key Informant Interview Guide and Direct Household Observation Checklist

1. Focus Group Discussion (FGD) Facilitator Guide

Objective: This focused group discussion guide is prepared to characterize locally available, underutilized, and climate-resilient crops for food complementary food.

Before the starting:

- Make sure the meeting space is ventilated and no disturbing noises.
- Make sure the participants are feeling safe
- Ensure the note taker is ready and sound recorder is working

During the discussion:

- Introduce the participants each other. Explain the objective of the discussion and confidentiality of the discussion points.
- Ensure that any participants can leave the focused group discussions at any time.
- Ensure verbal informed consent to participate in the focused group discussion.
- Thank the participants for their willingness to participate

After the discussion:

- Allow the participants to ask any question they have about the discussion.
- Debrief together (facilitator, note taker and if applicable interpreter) and write up any additional information as soon as possible so that it is not forgotten.

The focused group discussion descriptions

| 1. | Focus Group number |  |
| --- | --- | --- |
| 2. | Date of Focus Group Discussion |  |
| 4. | Meeting place (Kebele, Woreda) |  |
| 5 | Moderator’s name |  |
| 6 | Note-taker’s name |  |

| Participant’s code | Age | | Sex  F= Female  M= Male | Education | Marital Status  1=Single  2=Married  3=Divorced  4=Widowed  5=Other, specify___ | Consent form signed  1= Yes  2= No |
| --- | --- | --- | --- | --- | --- | --- |
| P1 |  | |  |  |  |  |
| P2 |  | |  |  |  |  |
| P3 |  | |  |  |  |  |
| P4 |  | |  |  |  |  |
| P5 |  | |  |  |  |  |
| P6 |  | |  |  |  |  |
| P7 |  | |  |  |  |  |
| P8 |  | |  |  |  |  |
| P9 |  | |  |  |  |  |
| P10 |  | |  |  |  |  |
| P11 |  | |  |  |  |  |
| P12 |  | |  |  |  |  |
| Name/Code of the Zone | |  | | | | |
| Name/Code of the Woreda | |  | | | | |
| Name/Code of the Kebele | |  | | | | |
| Name of facilitator: | |  | | | | |

Focused Group Discussion Guiding Questions

Introduce yourself to the group and ask the group the introduce themselves. Then explain that the topic you want to discuss today is to characterize locally available, underutilized, and climate-resilient crops for food complementary food.

| Q1. What is your current occupation? |
| --- |
|  |
| Probe: Type and duration away from the infant and young child |
| Q2. Can you describe the benefit of breastfeeding your child? |
|  |
| Probe: In terms of duration and frequency |
| Q3. When did you give complementary food for your child? If it’s before 6 months why? |
|  |
| Q4. Can you describe the type of food you use to prepare complementary food? |
|  |
| Probe: The varieties of foods and frequency of each food item usually used for your child |
| Q5. How frequently do you feed complementary food to your child? |
|  |
| Probe: in terms of frequency in a day and/or week |
| Q6. Can you describe the food items available in the community used for complementary food? |
|  |
| Q7: Which food item is available through the year? |
|  |
| Probe: Which food items are also available in what season? |
| Q8. What are the challenges you usually face to prepare complementary foods? |
|  |
| Q9. Do you think the challenges are similar across different seasons? |
|  |

Reminder: If all the option are being exhausted you thank the group for their time and information.

End the meeting.

1. Key Informant Interview Guide

Introduction: Hello, this is ………... I am a Human Nutrition PhD student at Hawassa. Currently I am working on a research work entitled Characterization of complementary feeding practice and locally available climate-resilient crops for complementary food among agro-pastoralists of Ethiopia. As a part of the project, I am identifying locally available climate-resilient crops and to develop complementary foods intended for infants and young children (6-23 months of age). I believe the work will have importance in promoting the climate resilient crops to improve the infant and young children nutrition in the community.

Thus, I am particularly interested in hearing about your views and opinions about the complementary feeding practices, climate resilient crops, and their role in mitigating the seasonal food insecurity.

Informed consent: The information gathered from this interview will be kept confidential and used only for this research purpose. I want to let you know that the interview will be recorded and transcribed. I would like to assure you that the results from interviews will be reported without mentioning the personal data.

With this I would like to ask your willingness to be interviewed and record the interview.

Is it ok to begin the interview?

Yes_______

No_______

If yes, continue

Key informant guiding questions

1. What is your educational background and current role in the woreda?
2. How long have you been working in the (mention his/her role) woreda/kebele?
3. What is your educational background?
4. Do you have a specific roles related to child nutrition? If yes, please probe to mention and describe.
5. Can you please give an insight about the any type of food available for infants and young child in the community? Probe for lists
6. What do you think the complementary feeding practices in the community?
7. How the food items listed are available throughout the year?
8. Can you describe the commonly food item usually consumed during dry and wet seasons?
9. What challenges do you see related to complementary feeding practices in the community? Probe for the challenges related to seasonality of food items.

Thank the Key Informant for their time!

1. Direct Observations Checklist
2. Types of complementary food given

Note: List all the foods that is considered by the women

1. Complementary food preparation steps followed

Note: Write all the steps followed to prepare the complementary food

1. List of food items/ingredients included in the Complementary food.
